# Discordant and Converting Receptor Expressions in Brain Metastases from Breast Cancer: MRI-Based Non-Invasive Receptor Status Tracking

**DOI:** 10.3390/cancers15112880

**Published:** 2023-05-23

**Authors:** Alexander Heitkamp, Frederic Madesta, Sophia Amberg, Schohla Wahaj, Tanja Schröder, Matthias Bechstein, Lukas Meyer, Gabriel Broocks, Uta Hanning, Tobias Gauer, René Werner, Jens Fiehler, Susanne Gellißen, Helge C. Kniep

**Affiliations:** 1 Department of Diagnostic and Interventional Neuroradiology, University Medical Center Hamburg-Eppendorf, Martinistraße 52, 20246 Hamburg, Germany; 2 Department of Computational Neuroscience, University Medical Center Hamburg-Eppendorf, Martinistraße 52, 20246 Hamburg, Germany; 3 Department of Radiotherapy and Radiation Oncology, University Medical Center Hamburg-Eppendorf, Martinistraße 52, 20246 Hamburg, Germany; 4 Center for Biomedical Artificial Intelligence (bAIome), University Medical Center Hamburg-Eppendorf, Martinistraße 52, 20246 Hamburg, Germany

**Keywords:** metastatic cancer, brain metastases, receptor status, machine learning, radiomics

## Abstract

**Simple Summary:**

Discordance and conversion of receptor expressions in metastatic lesions and primary tumors is often observed in patients with brain metastases from breast cancer. Personalized therapy requires continuous monitoring of receptor expressions and dynamic adaptation of applied targeted treatment options. This study sought to evaluate if quantitative MR image features can predict the receptor status of brain metastases from breast cancer using machine learning algorithms. Results indicate that receptor status can be differentiated non-invasively based on routine MR imaging data. The proposed approach could allow non-invasive expression tracking at high frequencies, and may support dynamic treatment optimization for personalized therapies.

**Abstract:**

Discordance and conversion of receptor expressions in metastatic lesions and primary tumors is often observed in patients with brain metastases from breast cancer. Therefore, personalized therapy requires continuous monitoring of receptor expressions and dynamic adaptation of applied targeted treatment options. Radiological in vivo techniques may allow receptor status tracking at high frequencies at low risk and cost. The present study aims to investigate the potential of receptor status prediction through machine-learning-based analysis of radiomic MR image features. The analysis is based on 412 brain metastases samples from 106 patients acquired between 09/2007 and 09/2021. Inclusion criteria were as follows: diagnosed cerebral metastases from breast cancer; histopathology reports on progesterone (PR), estrogen (ER), and human epidermal growth factor 2 (HER2) receptor status; and availability of MR imaging data. In total, 3367 quantitative features of T1 contrast-enhanced, T1 non-enhanced, and FLAIR images and corresponding patient age were evaluated utilizing random forest algorithms. Feature importance was assessed using Gini impurity measures. Predictive performance was tested using 10 permuted 5-fold cross-validation sets employing the 30 most important features of each training set. Receiver operating characteristic areas under the curves of the validation sets were 0.82 (95% confidence interval [0.78; 0.85]) for ER+, 0.73 [0.69; 0.77] for PR+, and 0.74 [0.70; 0.78] for HER2+. Observations indicate that MR image features employed in a machine learning classifier could provide high discriminatory accuracy in predicting the receptor status of brain metastases from breast cancer.

## 1. Introduction

The occurrence of brain metastases substantially impacts prognosis and health-related quality of life in patients with breast cancer [1]. For breast cancer, reported incidences for cerebral metastases range between 10–30% [2,3,4]. In breast cancer therapy, estrogen receptor alpha (ER), progesterone receptor (PR), and human epidermal growth factor receptor 2 (HER2) status of the primary tumor have become the major determinants of targeted treatment regimens [5]. Moreover, in the case of metastatic disease, therapeutic decisions are often based on the receptor expression of the primary lesion. Biopsies are seldom performed, and are only considered if the metastases’ locations and the patient’s general health condition allow invasive biopsy procedures. Several studies have demonstrated that metastases have a substantial tendency for mutations in receptor expression, leading to discordant and diverging receptor status [6,7,8,9,10,11,12,13,14]. A recent meta-analysis reported the following rates for receptor conversion in patients with metastatic breast cancer by primary tumor receptor expression: For ER, PR, and HER2, the pooled positive to negative conversion rates were 22.5%, 49.4%, and 21.3%, respectively; negative to positive conversion rates were 21.5%, 15.9%, and 9.5% [7]. Following these findings, several guidelines included recommendations for biopsies of metastases to confirm the metastases’ receptor expressions [5]. Moreover, recent updates extend this approach, and also recommend reassessments of metastatic receptor expressions if clinically feasible [1]. However, for cerebral metastases, biopsies are associated with risks of hemorrhage, infection, and neurologic complications, or are technically not feasible [7]. 

For these patients, non-invasive advanced radiological techniques may allow receptor status tracking at high sampling rates at low risk and cost. Such an approach could enable continuous monitoring of receptor expressions and dynamic adaptation of applied targeted treatment options. The present study investigates the potential of receptor status determination via a machine learning approach. We hypothesize that quantitative imaging biomarkers of brain metastases extracted from standard-of-care multispectral MR image data can be used to non-invasively determine the receptor status of these metastatic lesions.

## 2. Materials and Methods

This single-center retrospective study based on anonymized data was approved by the local ethics committee and the requirement for patient consent was waived (WF-144/20). The study was conducted in accordance with the Declaration of Helsinki. The data supporting the results of the analysis are available from the corresponding author upon reasonable request.

**Figure 1:**
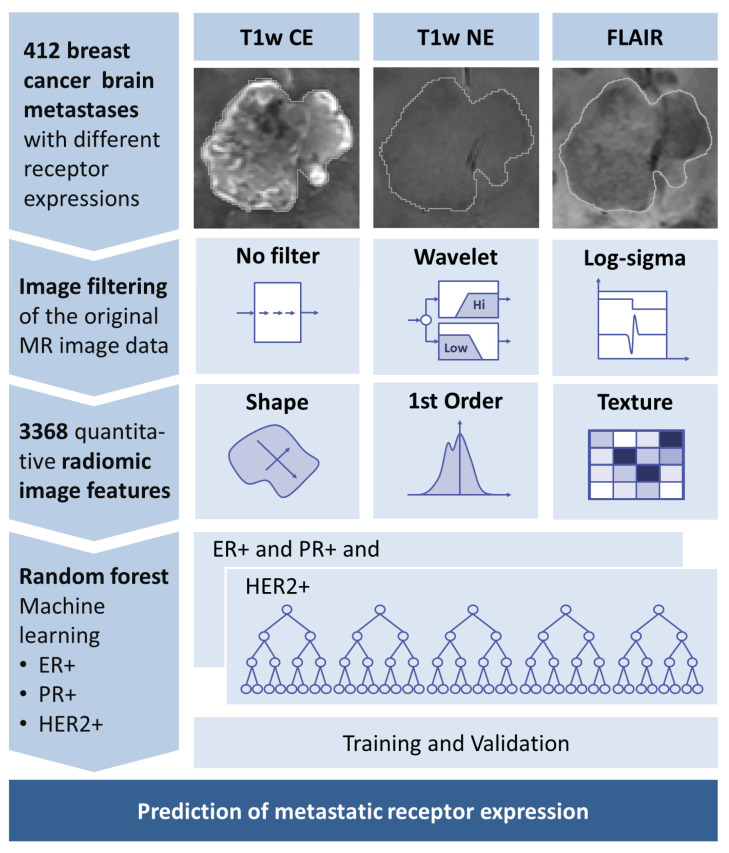
Overview of proposed algorithm for receptor status prediction. T1w CE, contrast-enhanced T1-weighted MR images; T1w NE, non-contrast-enhanced T1-weighted MR images; ER, estrogen receptor; PR, progesterone receptor; HER2, human epidermal growth factor receptor 2.

An overview of the proposed algorithm for receptor status determination is given in Figure 1; its modules are described in the following.

### 2.1. Patients

**Figure 2:**
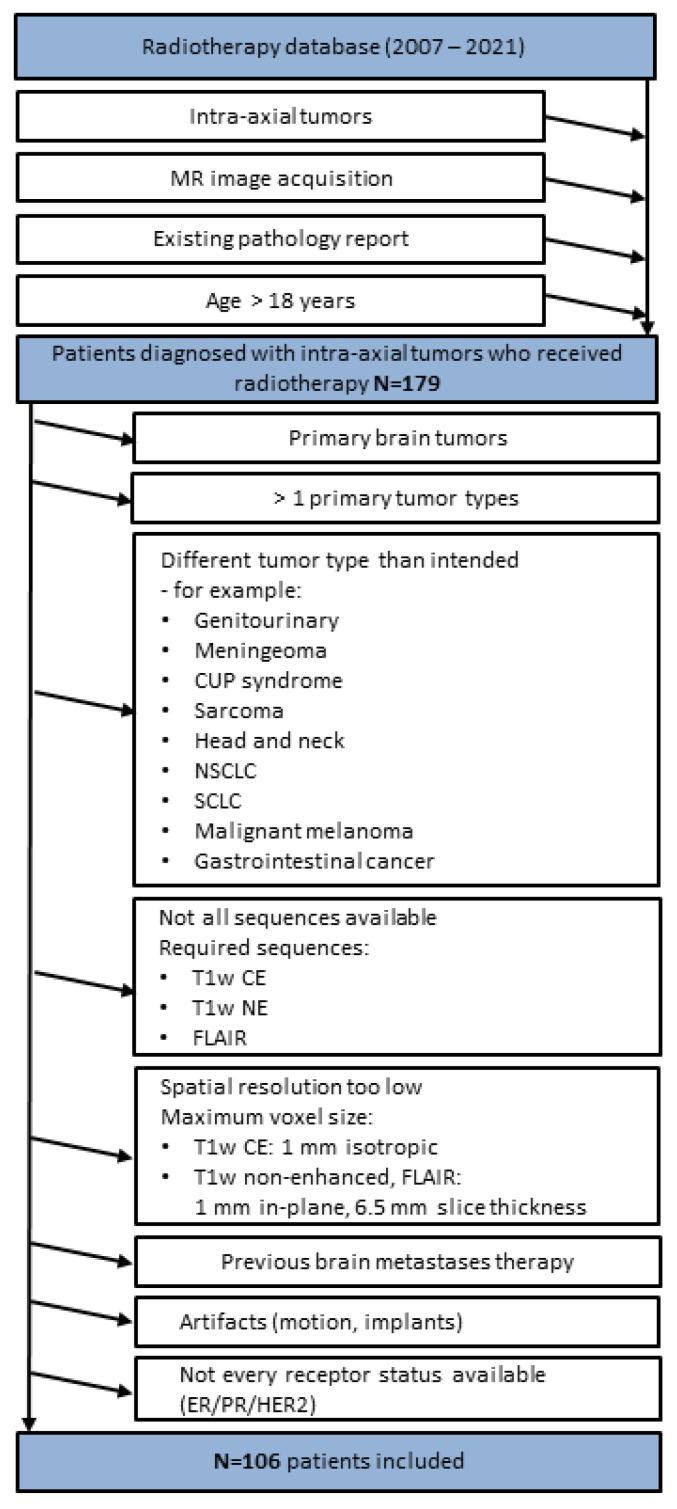
Patient inclusion and exclusion criteria flow chart. T1w, T1-weighted; CE, contrast-enhanced; NE, non-contrast-enhanced; FLAIR, non-contrast axial-fluid-attenuated inversion recovery; CUP, cancer of unknown primary; NSCLC, non-small cell lung cancer; SCLC, small cell lung cancer; ER, estrogen receptor; PR, progesterone receptor; HER2, human epidermal growth factor receptor 2.

MR imaging studies over a 14-year period (September 2007–September 2021) were retrospectively screened for the presence of brain metastases based on availability sampling. Clinical parameters were collected through chart review. Inclusion criteria (Figure 2) were defined as follows: (a) intra-axial tumors; (b) MR image acquisition; (c) existing pathology report; (d) age > 18 years. Subsequently, the patients were further screened. Exclusion criteria (Figure 2) were defined as follows: (a) primary brain tumor; (b) >1 primary tumor type; (c) other tumor type than breast cancer; (d) not all MRI sequences available; (e) spatial resolution too low; (f) previous metastasis-specific therapy; (g) artifacts; (h) not every receptor status available. Included images were acquired during the initial diagnostic procedure and before specific therapies for the brain metastases were initiated. In total, 412 brain metastases from 106 patients (mean age 56 year, range 33–84 year; 103 females, 3 males) were included (Table 1). Extracted patient data included type of primary neoplasm, receptor status, patient sex, and patient age. In order to avoid a clumping effect with regard to the feature expression and machine learning model training, the number of metastases per patient was limited to the 10 largest lesions. We obtained the exact tumor classification from the pathology department of our hospital from the tissue biopsies of the primary tumor side or of the metastases. For the classification of receptor status, our laboratory works according to the AWMF guidelines. Thus, a negative ER/PR receptor status occurs when there are less than 1% positive tumor cells, a low positive ER/PR status occurs when there are 1–9% positive tumor cells, and a positive status occurs when there are 10% or more than 10% positive tumor cells in the tissue examined [15,16]. Regarding our study, we considered a low positive status as the threshold for ER+/PR+. HER2 positivity is indicated by a uniformly intense circular membrane response of more than 10% (=3+) of tumor cells during immunohistochemistry [17], or a preferably detected gene amplification by in situ hybridization (ISH). Of the 106 patients included, metastasis-specific receptor status was known in 38 patients. In the remaining collective, the receptor status of the primary tumor was used. 

### 2.2. Image Acquisition

Images were acquired with 1.5 Tesla scanners (Magnetom^®^ Symphony, Magnetom^®^ Avanto, and Magnetom^®^ Sonata, all manufactured by Siemens Healthcare, Erlangen, Germany) or 3 Tesla scanners (Ingenia^®^, Philips Medical Systems, Best, The Netherlands; Magnetom^®^ Skyra and Magnetom^®^ Vida, Siemens Healthineers AG, Erlangen, Germany). Since some MRI studies were acquired in external clinics, the scanners could not be traced for all included patients. Employed imaging protocols included non-enhanced axial T1-weighted (T1w NE) spin echo with flow compensation and non-contrast axial-fluid-attenuated inversion recovery (FLAIR). After weight-adjusted injection of a gadolinium-based contrast agent (0.1 mmol/kg), contrast-enhanced axial T1-weighted spin echo sequences with flow compensation or three-dimensional T1w gradient echo sequences were acquired (T1w CE). 

### 2.3. Tumor Segmentation

Tumor delineation was performed based on the original T1w CE scans using a semi-automatic approach facilitated by ITK-SNAP software (Biomedical Imaging Resource; version number 3.8.0; Penn Image Computing and Science Laboratory University of Pennsylvania (PA), Scientific Computing and Imaging Institute University of Utah (UT)) and controlled by two radiologists (SG, 13 years clinical experience in diagnostic neuroradiology in an academic full-service hospital; HK, 2 years clinical experience in diagnostic neuroradiology in an academic full-service hospital). Regions of interest (ROIs) were defined according to the visible gross tumor volume; readers were blinded to all clinical data. Consensus ROIs were calculated based on overlapping segmentations when inconsistencies occurred.

### 2.4. Image Post-Processing

**Figure 3:**
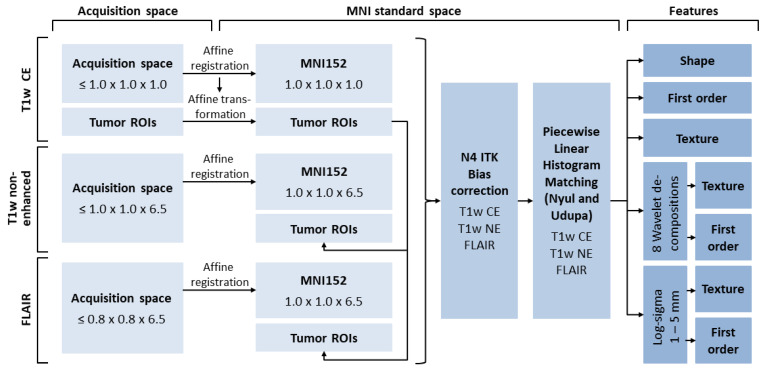
Image processing pipeline. Spatial resolutions are shown in mm. MNI, Montreal Neurological Institute of McGill University Health Centre; T1w, T1-weighted; CE, contrast-enhanced; NE, non-enhanced; FLAIR, non-contrast axial-fluid-attenuated inversion recovery; ROI, region of interest.

In order to extract ROIs from all acquired sequences and to normalize texture metrics, the bone mass was removed from all images with ROBEX software (NITRC; University of Massachusetts Medical School in Worcester, MA) [18], and all images were registered to a common standard space (MNI152 [19]). To further reduce bias in texture analysis, spatial resolutions of target spaces for image registration were selected so that no upsampling of image data was necessary. Hence, images were registered to atlases resliced to the maximum voxel dimensions of each spatial dimension in the respective unprocessed original data. Registration was conducted with NiftyReg (Centre for Medical Image Computing; University College London, UK) [20]. To account for non-standardized intensity calculations of MR scanners, all images were N4ITK bias-corrected [21] and piecewise linear histogram matched [22]. Tumor segmentations were transformed into MNI standard space employing transformation matrices obtained from image registration. Two MDs (HK and SG) visually verified the success of image post-processing. An overview of the image post-processing steps is given in Figure 3.

### 2.5. Feature Extraction

Texture- and intensity-based analyses require a certain minimum number of included voxels to ensure meaningful marker results. Hence, only features from metastatic lesions fulfilling the following criteria were included in the analysis: (a) metastasis volume equal to or greater than 100 mm³, equating to a cube with an edge length of 4.6 mm; (b) metastasis belongs to the 10 largest metastases per patient. The total number of included metastases was n = 412 (Table 1). Extracted radiomic features were defined according to the employed PyRadiomics Python package v2.2.0 [23]. For each analyzed MR sequence (T1w CE, FLAIR, T1w non-enhanced), extracted features comprised 252 first-order features (18 based on the unfiltered images, 144 wavelet decompositions, 90 log-sigma Laplacian of Gaussian-filtered images), 952 texture features (68 based on the unfiltered images, 544 wavelet decompositions, 340 log-sigma Laplacian of Gaussian-filtered images), and 14 shape features (based on the unfiltered images). Since some features in some sequences could not be calculated from the image properties, these features were excluded from further consideration, resulting in a total number of 3368 image features and the patient age.

### 2.6. Machine Learning

Machine-learning-based classification of receptor status was performed using random forest algorithms implemented in the Python scikit-learn environment v1.1.1 [24]. Random forest classifiers have a comparably low tendency to overfit [25,26], and support classification tasks for data sets comprising numerous and heterogeneous predictors. Furthermore, random forest classifiers allow analysis of cluster-correlated observations, e.g., patients with multiple metastases [27]. Three different classifiers were trained for differentiation of the following cases: Classifier 1, hormone/estrogen-receptor-positive vs. -negative; Classifier 2, hormone/progesterone-receptor-positive vs. -negative; Classifier 3, human epidermal growth factor receptor 2 (HER2)-positive vs. -negative. Triple-negative status can be assumed for ER-, PR-, and HER2-negative metastases.

### 2.7. Feature Selection

Predictive values of the employed image features were calculated separately for each training data set based on Gini impurity measures [28]. Since many features are expected to be correlated, e.g., due to using several filters on the same image, and owing to the risk of overfitting the model with too many features, we restricted the number of features used to the 30 most important features [29]. For all model evaluation runs, feature importance was determined based on the respective training data only.

### 2.8. Model Hyperparameters, Training, and Testing

The number of trees was set to random forest classifier default. The square root of the total number of features utilized for training and prediction was used for setting the number of features per node according to the literature [25,30]. In total, 10 randomly permuted 5-fold cross-validation sets [31,32] generated from the n = 412 samples were used for model evaluation. All cross-validation sets were randomly drawn with stratification for receptor status. In addition, to prevent bias from cluster correlation, metastases of patients with multiple lesions were either assigned to the validation or to the training set. Predictive performance was evaluated based on all validation/test sets, each set containing n = 329 training samples and n = 83 validation/test samples. 

### 2.9. Statistics

Receiver operating characteristic (ROC) curves of the training/validation sets were calculated based on cross-validation set means. Hence, ROC curves from cross-validation may serve as unbiased estimates for model classification performance in a generalized setting. Statistical significance of the areas under the curves (AUCs) was assumed if *p*-values were < 0.05 in all cross-validation sets. Model prediction instability (i.e., standard deviation of ROC curves) was evaluated using 10 randomly drawn 5-fold cross-validation sets. *p*-values were calculated with Mann–Whitney/Wilcoxon U statistics using the verification v1.42 R package [33]. Classifier performance was evaluated through ROC AUC metrics. Furthermore, the classifiers were analyzed at operating points according to the maximum value of the Matthews correlation coefficient (*MCC*) [34]. The *MCC* evaluates all fields of the confusion matrix, and is considered as a favorable measure for unbiased comparisons of binary classifiers [35]. Incorporating *TP*s (true positives), *TN*s (true negatives), *FP*s (false positives), and *FN*s (false negatives), *MCC* is defined as

$$MCC=\frac{TP\;\cdot \;TN-FP\;\cdot \;FN\;}{\sqrt{\left(TP+FP\right)\left(TP+FN\right)\left(TN+FP\right)\left(TN+FN\right)}}$$

Confidence intervals for classification performance metrics were calculated using pROC v1.18.0 [36], ThresholdROC v2.9.0, and psychometric v2.3 R packages, and the Python scikit-learn metrics environment v1.1.1 [24]. 

## 3. Results

The analysis is based on T1w CE, FLAIR, and T1w NE MR images of 412 brain metastases from breast cancer. The training set includes n = 329 samples; the independent validation set includes n = 83 samples in 1 out of 10 randomly drawn 5-fold cross-validation sets.

### 3.1. ER Receptor

**Figure 4:**
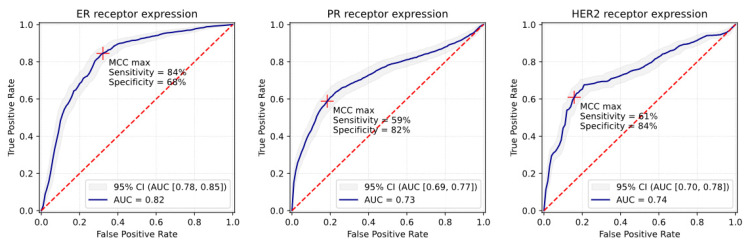
Receiver operating characteristic (ROC) curves and Matthews correlation coefficient (MCC) cut-off point analysis of ER+/ER+ and HER2+ classifiers. Training/validation: 5-fold cross-validation, consideration of cluster correlation, sample size of 329 metastases. Independent test/validation set: sample size of 83 metastases. Crosses depict the maximum MCC cut-off points. ER, estrogen receptor; PR, progesterone receptor; HER2, human epidermal growth factor receptor 2; AUC, area under the curve; CI, confidence interval.

ROC AUC in the validation sets for predicting the receptor status based on multiparametric MR scans was 0.82 with 95% CI [0.78; 0.85] (Figure 4). At the maximum MCC cut-off point, the classifier yielded specificities of 68% (95% CI [65%; 71%]) at sensitivities of 84% (95% CI [81%; 88%]) (Figure 4, Table 2). Furthermore, the positive predictive value (PPV) and negative predictive value (NPV) were 69% and 84%, respectively; accuracy was 76% and MCC was 0.53 (Table 2).

### 3.2. PR Receptor

ROC AUC of the validation sets was 0.73 with 95% CI [0.69; 0.77] (Figure 4). At the maximum MCC cut-off point (MCC = 0.42), specificities of 82% (95% CI [77%; 85%]) at sensitivities of 59% (95% CI [56%; 64%]) were observed (Figure 4, Table 2). PPV and NPV were 69% and 74%, respectively; accuracy was 72% (Table 2). 

### 3.3. HER2 Receptor

ROC AUC of the validation sets was 0.74 with 95% CI [0.70; 0.78] (Figure 4). At the maximum MCC cut-off point (MCC = 0.47), specificities of 84% (95% CI [81%; 88%]) at sensitivities of 61% (95% CI [57%; 65%]) were observed (Figure 4, Table 2). PPV was 73%, NPV was 76%, and accuracy was 75% (Table 2). 

### 3.4. Triple-Negative

Triple-negative status can be assumed for metastases with PR-, ER-, and HER2-negative predictions.

Comparative analysis of randomly drawn five-fold cross-validation sets confirmed low variability of results, hence indicating stable model performance. ROC AUCs of the independent validation/test sets were >0.5 with *p*-value < 0.05 for all three classifiers.

### 3.5. Feature Importance

**Figure 5:**
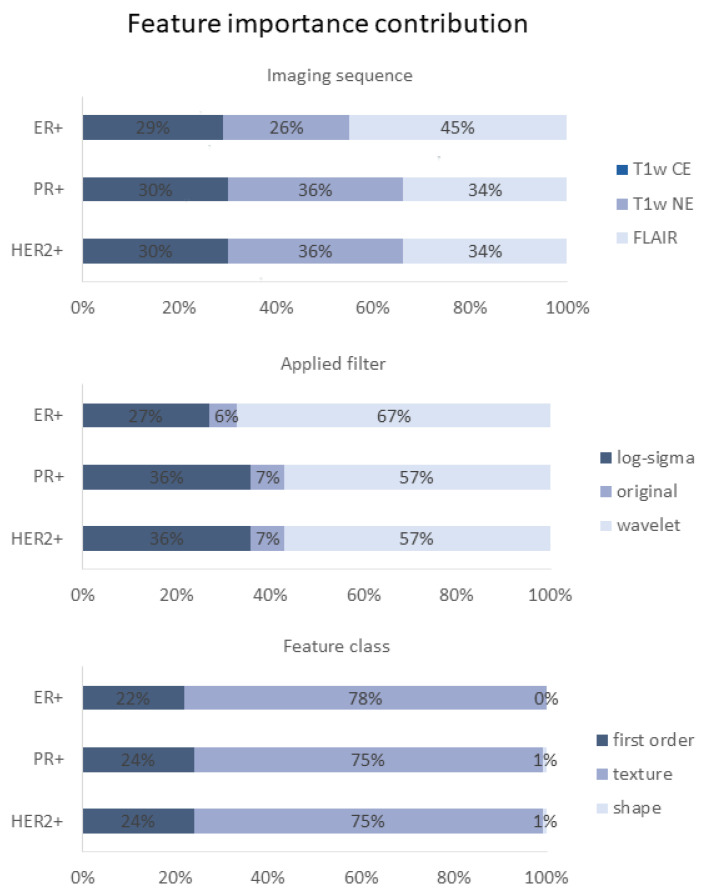
Radiomic feature distributions of top 100 ranked most important predictors for HER2+ and ER+/PR+ classifiers. Numbers show shares of total feature importance contribution. ER, estrogen receptor; PR, progesterone receptor; HER2, human epidermal growth factor receptor 2; T1w, T1-weighted; CE, contrast-enhanced; NE, non-enhanced; FLAIR, non-contrast axial-fluid-attenuated inversion recovery.

Mean importance numbers generated from all training sets were used for the feature importance analysis. While PR+ and HER2+ classifiers employed mainly T1w NE images (36% of total predictive importance), ER+ classification was mostly FLAIR-dependent (45% of total predictive importance). For T1w CE (30%), a lower contribution to predictive performance was observed in PR+ and HER+ classifiers, respectively; 29% was observed for the ER+ classifier (Figure 5). Overall, however, the different image sequences have similar proportions of the feature importance distribution, especially with regard to PR+ and HER2+. Top 100 ranked features differed between ER+ and PR+/HER2+ classifiers, indicating the presence of specific predictive imaging marker signatures for ER+ classifiers and similar predictive imaging markers for PR+ and HER2+ classifiers. In total, only 2 identical image features ranked among the top 100 most important predictors for all 3 receptor classes, but considering only PR+ and HER2+ receptor classes, they share 98 of the 100 most important image features. With regards to applied filters, the shares in feature importance contribution for ER+ and PR+/HER2+ classifiers were as follows: Log-sigma-filtered images, 27% vs. 36%; wavelet-filtered images, 67% vs. 57%; and unfiltered original images, 6% vs. 7% (Figure 5). Differentiated by feature classes, the ER+ classifier utilizes more texture features (78%) compared to the PR+/HER2+ classifiers (75%). Shape features were not ranked among the top 100 predictive markers for the ER+ classifier, and had only little impact in PR+/HER2+ classification (Figure 5). Overall, the lowest-performing image sequence was T1w CE, the lowest-performing feature class was shape, and including log-sigma and wavelet filters in the analysis seems to be important (Figure 5).

**Figure 6:**
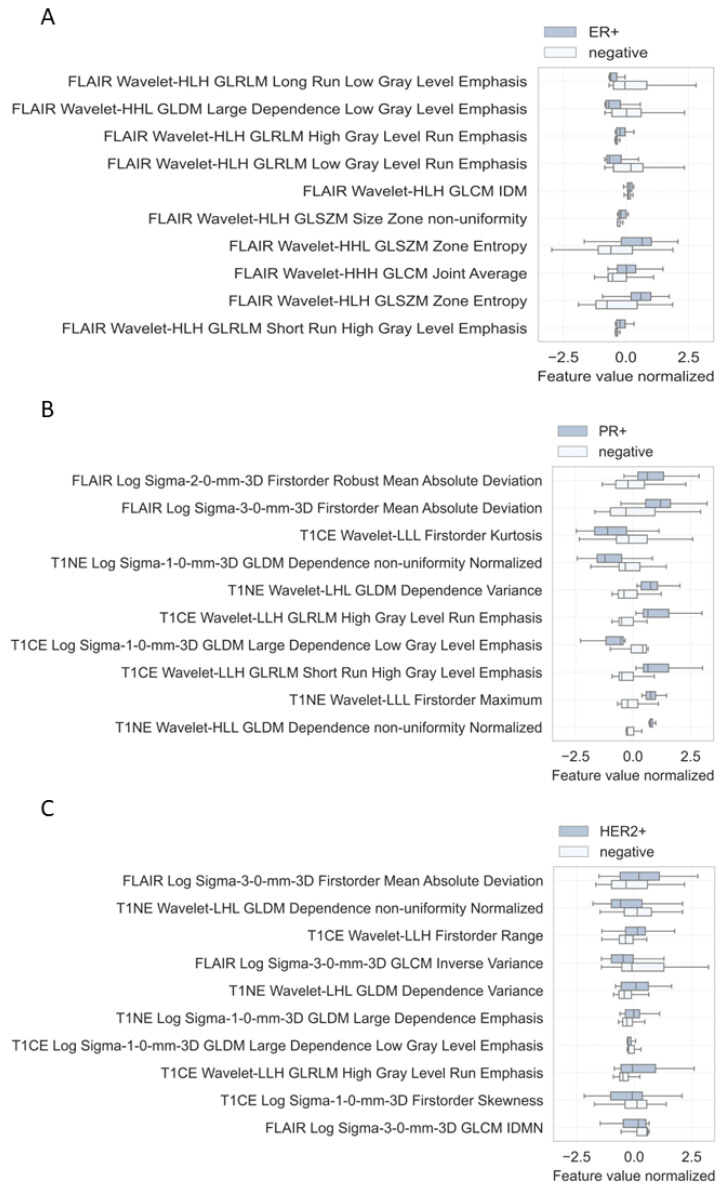
Radiomic signatures of 10 most important image features for the ER+ classifier (**A**), PR+ classifier (**B**), and HER2+ classifier (**C**). Comparison of respective receptor-positive metastases with receptor-negative metastases. T1NE, T1 non-enhanced; T1CE, T1 contrast-enhanced; FLAIR, non-contrast axial-fluid-attenuated inversion recovery; H, high-pass wavelet decomposition; L, low-pass wavelet decomposition.

Figure 6 shows boxplots of the top 10 normalized feature values for the ER+ classifier (A), PR+ classifier (B), and for the HER2+ classifier (C). The radiomic signature value distribution suggests that ER+ metastases have lower gray level emphasis compared to hormone-receptor-negative metastases; however, zone entropy is more pronounced in ER+ metastases, but both zone entropy predictors exhibit large variability for the same predictor set, and might not allow for stable classification results (Figure 6A). Figure 6B,C confirm similar predictor distributions for PR+ and HER2+ metastases, but the overall picture of the distributions of individual predictors is more heterogeneous compared to the ER metastases. Still, different characteristics of gray level emphasis seem to be important in PR+ prediction as well. The first-order mean absolute deviation appears to be much higher for PR+ and HER2+ metastases than for negative receptor status, exhibiting large variability for the same predictor set. All differences in mean feature values between receptor-positive and -negative metastases were statistically significant (*p*-value < 0.05).

## 4. Discussion

We proposed to use quantitative image features from standard multiparametric MR scans to determine the receptor status of brain metastases in patients with metastatic breast cancer without invasive procedures. Our results show that machine learning algorithms can predict the receptor status, achieving AUCs of 0.82 for ER+ metastases, 0.73 for PR+ metastases, and 0.74 for HER2+ metastases. The study demonstrates that an artificial intelligence approach has the potential to non-invasively track receptor expression in patients with metastatic breast cancer.

Over the past few years, advances in targeted treatment options for patients with primary and advanced breast cancer have made receptor status expression of the primary tumor a leading determinant of therapeutic regimes, and have led to a significant improvement in outcomes [1,37,38]. However, as indicated above, in patients with metastatic breast cancer, diverging and discordant receptor status are observed with the following frequencies: 19.3% for ER, 30.9% for PR, and 10.3% for HER2 expression [7]. Moreover, studies have shown that receptor expressions can diverge even for different metastases in the same patient [8]. Receptor divergence might have significant impact in both of the two possible scenarios: In the case where the receptor expression is lost, patients will receive their now-ineffective targeted therapy at the price of related adverse effects and additional costs for the medication. In the case of mutations leading to receptor expression gain, patients will not be prescribed respective medications and will not benefit from effective targeted treatment options. However, to date, there is no randomized, controlled, trial-based evidence for improved survival in patients with therapy adaptations according to their metastases’ receptor status [1]. Nevertheless, in order to bridge this evidence gap and to ensure the best possible patient care, recent guidelines recommend (a) to assess and to retest receptor status of the metastatic lesion at least once [5,39], and (b) to align therapeutic regimes according to the receptor expression of the metastatic lesion [5], or to consider any positive receptor expression from metastases or primary tumor biopsies [1]. Still, biopsies and rebiopsies can only be conducted if the respective lesion is accessible and adverse effects are expected at appropriate rates considering the potential benefits of therapy adjustments. Especially in patients with brain metastases, both of these preconditions are often difficult to evaluate and are seldom fulfilled. Hence, the here-proposed method for non-invasive receptor status determination based on imaging biomarkers derived from standard-of-care MR brain scans might (a) improve therapeutic management by allowing receptor status monitoring at high frequencies and enabling agile therapy optimization, (b) enhance the general understanding of receptor divergence mechanisms, and (c) generate further evidence with respect to the effects of receptor divergence and applied targeted treatment regimens on overall survival.

Several studies have investigated approaches for non-invasive receptor status diagnosis in breast cancer. However, most analyses were limited to primary tumor characteristics: Catalano et al. (2017) [40] show correlations of breast cancer phenotype with imaging markers from PET-MR scans, with cross-validation accuracies of 38% to 48% in a multivariate analysis. Incoronato et al. (2018) [41] also utilize PET-MR imaging biomarkers, and report multivariate model accuracies of 78%; however, cross-validation was not performed, and results might worsen significantly in a training/validation approach. With ROC AUCs of 0.62 to 0.65, a large MR-imaging-based analysis by Saha et al. (2018) [42] reported moderate associations of molecular subtypes with imaging biomarkers. 

To date, only a few approaches for non-invasive metastases receptor expression diagnosis have been investigated. An analysis related to the receptor status of breast cancer metastases in the brain was conducted by Luo et al. (2022) [43]: A logistic regression model was trained to predict the receptor status of brain metastases. The predictions were compared to pathology reports of the metastases and primary tumors. The study reported AUCs of 0.89, 0.88, and 0.87 for ER+, PR+, and HER2+, and receptor discordance between metastasis and primarius of 27.5% for ER+/PR+ and 5% for HER2+. A training and a test set were used, but cohorts were relatively small. Another approach by Cao et al. (2022) [44] investigated whether habitat-based radiomics was capable of predicting the EGFR mutation status in brain metastases from primary lung adenocarcinoma, with an AUC of 0.9 in the best-performing external validation model. Hohm et al. (2022) [45] found that, using standardized MR imaging parameters and epidemiologic data, it is possible to detect genetic differences (H3 K27M+/−) in pediatric diffuse midline gliomas. Thus, a better prognosis may be estimated and a more personalized therapy may be offered in the future. Liquid biopsy in metastasized breast cancer as the basis for treatment decisions was discussed by Krawczyk et al. (2016) [46]. Another non-invasive approach to distinguish cerebral tumor entities, from which receptor status prediction could also benefit, was investigated by Foda et al. (2022) [47] by examining different vascular parameters of the various tumors in MR imaging data. Nevertheless, as of today, all the approaches have not been translated into clinical routine.

The results of the training/validation analysis show superiority of the ER+ classifier (ROC AUC 0.82) compared to the PR+ and HER2+ classifiers (ROC AUC 0.73 and 0.74). Inferior results for predicting HER2 status are in line with primary tumor classification performances reported by other studies [42], suggesting the presence of more heterogeneous imaging characteristics in these lesions. A comparison of value distributions of the 10 most important histogram-based predictors employed by the ER+ classifier with value distributions of PR+ and HER2+ metastases supports this hypothesis: Respective predictors in PR+ and HER2+ lesions (Figure 6B,C) exhibit a substantial higher variability compared to ER+ tumors (Figure 6A). Furthermore, median values of positive and negative metastases are much closer together for PR+ and HER+ predictors than for ER+ metastases. This suggests inferior applicability of these high-predictive-power markers for differentiating receptor status in PR+ and HER2+ metastases, and might explain the discrepancy between selected feature sets between the three classifiers. FLAIR sequences seem to be more applicable for ER+ metastases (Figure 6A); therefore, ER+ metastases might form a specific edema-associated pattern, which can be recognized by the machine learning algorithm.

Although numerous associations between quantitative image features and molecular tissue characteristics have been demonstrated [48], radiomics-based artificial intelligence systems are still often criticized for potentially irreproducible and unintelligible decision paths. To address these concerns and establish ties to conventional image findings, we evaluated the employed image features with regards to possible interpretations in classic visual assessments. Our analysis shows that ER+ exhibit narrower value distributions and higher uniformity in wavelet-filtered images and FLAIR sequences, suggesting a more homogenous appearance of these lesions in conventional visual readings compared to hormone-receptor-negative ER+ and PR+/HER2+ metastases. Feature analysis for PR+/HER2+ metastases indicate higher image heterogeneity and more pronounced intensity edges. Top-performing predictors were similar, but often in the opposite expression direction. Triple-negative tumors were not studied separately, and due to the low overlap of shared top 100 predictors, it is difficult to draw conclusions about their radiomic signature in this study design. In line with our results, studies investigating MR imaging characteristics of primary breast tumors reported higher heterogeneity for triple-negative and HER2+ lesions compared to hormone-receptor-positive tumors [49,50]. Still, several of the observed characteristics diverge between the different analyses, and do not provide clear evidence.

Our study had general limitations of radiomic analyses, as previously reported [51,52,53,54]. Furthermore, the following study-specific limitations were observed: First, an expansion of the number of included patients would further increase the generalizability of the results. Small sample sizes are a general concern for radiomics analysis, and are due to the limited availability of standardized and annotated multicenter imaging data sets. Nevertheless, the conducted model stability assessment indicates sufficient robustness of results for the evaluation of the feasibility and limitations of imaging-marker-based receptor status predictions. Second, it cannot be excluded that some patients in this collective were already under systemic therapy during the development of the brain metastases. In these cases, therapy was not metastasis-specific like radiosurgery that would hypothetically lead to an alteration of radiomic signatures Third, we did not differentiate receptor expression by the 2015 St Gallen Consensus subtypes Luminal A, Luminal B, HER2 overexpression, and Basal-like [38,55]. Such classification would necessitate the inclusion of Ki67 prediction and a significantly more complex classifier design to account for the required multiclass approach. As the number of classes and available training sample sizes are major determinants of prediction performance, such an approach would either necessitate a substantial expansion of sample size or would potentially degrade classifier performance. Fourth, we defined ground truth for classifier training according to pathology reports of brain metastases if available, otherwise we used primary tumor biopsies. This was due to the fact that metastases-specific pathology reports were only available for 38 of the 106 included patients. In our subgroup analysis of patients for whom we had the receptor status of primarius and metastasis, a change in at least 1 receptor status (ER, PR, HER2) occurred in 8 of 38 patients, corresponding to a conversation rate of 21%. This rate is also in line with the current literature [7]. Assuming a total receptor conversion rate of 20%, respective wrongly defined ground truth in 20% of cases might partly explain the observed classification errors of 16% (ER+), 26% (PR+), and 24% (HER2+). Because the receptor status of each individual metastasis was often not determined in patients with multiple brain metastases, we used the status of the known metastasis as the ground truth in these cases. However, the notion that the status between individual metastases in the same patient is identical is not necessarily the case [8]. Nevertheless, in the subgroup of our patient collective (n = 5) in which the expressions of all metastases were known, they matched within patients. Fifth, low-resolution images were excluded from the study to improve the validity of the texture analysis. Inclusion of low-resolution images would potentially reduce prediction performance, as texture features substantially contribute to the model’s prediction power. Sixth, the conducted semi-automatic delineation of lesion ROIs still leaves a certain degree of human observer dependence within the automated process. Consensus ROIs were partly used to minimize its impact. Further, it was observed that variations in segmentations have a comparably small effect on quantitative image feature values [56,57].

Upcoming studies may investigate the full potential of artificial-intelligence-based receptor status prediction through a substantial enlargement of the study population, employment of standardized high-resolution images, comprehensive integration of only metastases-related histopathological reports, and the adaption of classifier design for the differentiation of subtypes according to the St Gallen Consensus. Moreover, future studies may explore other fields of application, including receptor status predictions for liver and bone metastases and adaption of the algorithm to other primary tumors. Once approved and translated into clinical routine, non-invasive receptor status determination could significantly contribute to optimized therapeutic regimes, and may improve outcomes for patients with metastatic cancer.

## 5. Conclusions

We hypothesized that quantitative image features extracted from standard-of-care multiparametric MR scans can be used for non-invasive determination of the receptor status of brain metastases in patients with metastatic breast cancer. Our results suggest that machine-learning-based algorithms can predict the receptor status, with AUCs of 0.82 for ER+ metastases, 0.73 for PR+ metastases, and 0.74 for HER2+ metastases. The observed narrow confidence intervals and low standard deviations of the ROC curves indicate high stability of predictive performance in the training/validation sets. Promising AUC metrics significantly > 0.5 in the independent test set confirm the initial hypothesis. Our study demonstrates that the proposed approach could allow non-invasive receptor expression tracking in patients with metastatic breast cancer.

Future studies could explore the full potential of artificial intelligence-based receptor status prediction in brain metastases of breast cancer patients and extend this approach to other fields of interest like liver or bone metastases and all together may improve outcomes for patients with metastatic cancer.

## Figures and Tables

**Table 1:** Demographic and clinical characteristics of the included metastases samples. The table shows number of included metastases, number of respective patients, age at the time of brain metastases diagnosis by receptor status, and number of metastases of the sample patient by receptor status. ER, estrogen receptor; PR, progesterone receptor; HER2, human epidermal growth factor receptor 2. * Number of patients included with single brain metastasis; ** number of patients included with multiple brain metastases.

	Number of		Age of Sample Patient			Number of Metastases of Sample Patient		
	Metastases	Patients	Mean	Min	Max	Mean	Min	Max
All patients	412	106 30 * 76 **	55.9	33	84	5.4	1	66
**Classifier 1 (ER)**	Total samples							
ER+	190	53	58.3	33	76	4	1	63
ER−	222	53	53.9	36	79	5.9	1	66
**Classifier 2 (PR)**	Total samples							
PR+	147	37	59.5	39	76	4.5	1	63
PR−	265	69	53.9	33	84	5.7	1	66
**Classifier 3 (HER)**	Total samples							
HER+	169	45	54.5	36	81	4.1	1	63
HER−	243	61	56.9	33	84	6.1	1	66

**Table 2:** Classification performance metrics of independent validation sets. Classification performance metrics of the machine learning classifiers. Classifier metrics are shown at cut-off points according to the maximum Matthews correlation coefficient. AUC, area under the curve; ER, estrogen receptor; PR, progesterone receptor; HER2, human epidermal growth factor receptor 2; PPV, positive predictive value; NPV, negative predictive value; MCC, Matthews correlation coefficient; CI, confidence interval.

		Matthews Correlation Coefficient (MCC) Maximum Operating Point					
Receptor Status	AUC [95% CI]	Sensitivity [95% CI]	Specificity [95% CI]	PPV [95% CI]	NPV [95% CI]	Accuracy [95% CI]	MCC [95% CI]
	5-fold cross-validation (n = 412 samples)						
**ER+**	0.82 [0.78; 0.85]	84% [81%; 88%]	68% [65%; 71%]	69% [67%; 72%]	84% [82%; 88%]	76% [73%; 78%]	0.53 [0.49; 0.58]
**PR+**	0.73 [0.69; 0.77]	59% [56%; 64%]	82% [77%; 85%]	69% [67%; 76%]	74% [70%; 76%]	72% [69%; 75%]	0.42 [0.37; 0.48]
**HER2+**	0.74 [0.70; 0.78]	61% [57%; 65%]	84% [81%; 88%]	73% [72%; 80%]	76% [74%; 78%]	75% [73%; 76%]	0.47 [0.45; 0.52]

## Data Availability

The data supporting the results of this study are available from the corresponding author upon reasonable request.
